# Epigenetic Biomarkers for Predicting Nucleoside Analog Drug Response and Resistance in Cancer

**DOI:** 10.3390/biom16040587

**Published:** 2026-04-15

**Authors:** John Kaszycki, Jackson C. Lin, Minji Kim, Hunmin Jung

**Affiliations:** The Division of Medicinal Chemistry, Department of Pharmaceutical Science, School of Pharmacy, The University of Connecticut, Storrs, CT 06269, USA

**Keywords:** nucleoside analogs, epigenetics, DNA methylation, histone modification, biomarkers, chemoresistance, precision oncology

## Abstract

Nucleoside analogs (NAs) play a central role in cancer therapy, either through direct cytotoxicity or epigenome reprogramming. They are clinically effective but have shortcomings in their long-term effectiveness because of variable patient responses and the emergence of resistance. There is growing evidence that DNA methylation, histone modifications, chromatin remodeling, and non-coding RNAs (ncRNAs) are key factors that determine sensitivity and resistance to NAs. This review summarizes existing evidence on the epigenetic control of cytotoxic and epigenetic nucleoside analogs, discusses predictive biomarkers of human Equilibrative Nucleoside Transporter 1 (hENT1) and deoxycytidine kinase (dCK) promoter methylation, histone modifications, and ncRNA signatures, and assesses the emerging strategies of multi-omic integration. Improvements in detection methods, such as high-resolution sequencing, single-cell profiling, and liquid biopsy, are addressed, along with the issues of reproducibility, tumor heterogeneity, and clinical translation. Epigenetic biomarkers are promising for patient stratification in clinical trials, although a lack of uniformity in technical and methodological approaches currently constrains their full potential. The future focus will be on standardized panels of biomarkers, real-time monitoring, rational combination strategies, and biomarker-directed clinical trial designs. Overall, epigenetic biomarkers are capable of changing nucleoside analog therapy into a more precise, durable, and personalized treatment approach.

## 1. Introduction

Nucleoside analogs (NAs) remain a crucial component of cancer chemotherapy and are employed in the treatment of the majority of solid tumors and hematologic malignancies [1]. Classical cytotoxic agents such as gemcitabine, cytarabine, fludarabine, 5-fluorouracil (5-FU), and 6-thioguanine (6-TG) can be incorporated into nucleic acids, leading to the disruption of nucleic acid synthesis, replication stress, and cell death [2]. More recently of interest are cytosine analogs with epigenetic action, such as azacitidine and decitabine, which epigenetically reactivate tumor suppressor genes by directly inhibiting DNA methyltransferases (DNMTs) [3]. While NAs are still the cornerstone of the therapeutic regimen, their overall efficacy over longer periods of time is diminished due to the variable individual responses and the occurrence of both intrinsic and acquired resistance [4,5]. The mechanisms of resistance consist of defective uptake and activation, rapid catabolism, and DNA repair, but are increasingly understood as epigenetically regulated [6,7]. Epigenetic changes such as DNA methylation, histone modifications, chromatin remodeling, and ncRNAs are unstable and dynamic gene expression modulators that impact various facets of NA pharmacology [4]. Epigenetic states affect therapeutic response through the regulation of drug transporters, metabolizing enzymes, and DNA repair mechanisms [8]. Unlike permanent genetic mutations, these reactive changes can both serve as targets for therapy and as markers of tumor adaptability to therapy. The establishment of stable epigenetic markers thus holds considerable promise for the prediction of nucleoside analog response, the possibility of patient stratification, and the bypassing of resistance [9]. The present review provides an overview of the understanding of epigenetic markers of nucleoside analog efficacy and resistance, examines the translational challenges, and explores their potential contribution to precision oncology.

## 2. Nucleoside Analogs: Mechanisms of Action and Resistance

### 2.1. Cytotoxic Nucleoside Analogs

Classic cytotoxic nucleoside analogs, such as gemcitabine, cytarabine, and fludarabine, are still front-line treatments for several hematological malignancies, such as acute myeloid leukemia (AML) and lymphomas, and for several solid malignancies like breast and pancreatic cancer [10]. They are effective therapeutically owing to their disruption of the process of replication metabolism of DNA and RNA. Following entry into the cell, they are converted into active triphosphate metabolites through the action of nucleoside kinases [11]. When activated, they will compete with normal endogenous nucleotides for incorporation into strands of DNA and RNA. NA incorporation leads to early termination of the chain, backbone distortion of the nucleic acid (e.g., distortions that inhibit elongation of the strand), and collapse of the replication fork. The resulting replication stress causes double-strand DNA breaks and activates DNA damage response mechanisms and mitotic catastrophe or apoptosis [12,13]. Recently, polymerase eta (polη) has been shown to be key to inserting NAs into DNA as well as counteracting replication stress by bypassing nucleotides through its intrinsic property of extremely low fidelity. For example, polη has been shown to both insert and bypass deoxyinosine triphosphate (dITP), inosine triphosphate (ITP) and 5-Fluro-2′-deoxyuridine triphosphate (5dUTP) with high efficiency [14,15,16].

Besides being incorporated into nucleic acids, cytotoxic nucleoside analogs also affect prominent enzymes of cellular metabolism (e.g., DNA polymerases, ribonucleotide reductase). Gemcitabine diphosphate is an effective inhibitor of the enzyme ribonucleotide reductase [17]. It depletes the intracellular store of deoxynucleotides for DNA synthesis and accelerates replication stress [18]. Cytarabine and fludarabine possess a bimodal action mechanism: both inhibit the synthesis of RNA and DNA and thus provide a cytocidal action [19]. Such complex processes justify their broad-spectrum anticancerous activity and their ability to overcome NA resistance [12,13].

**Figure 1:**
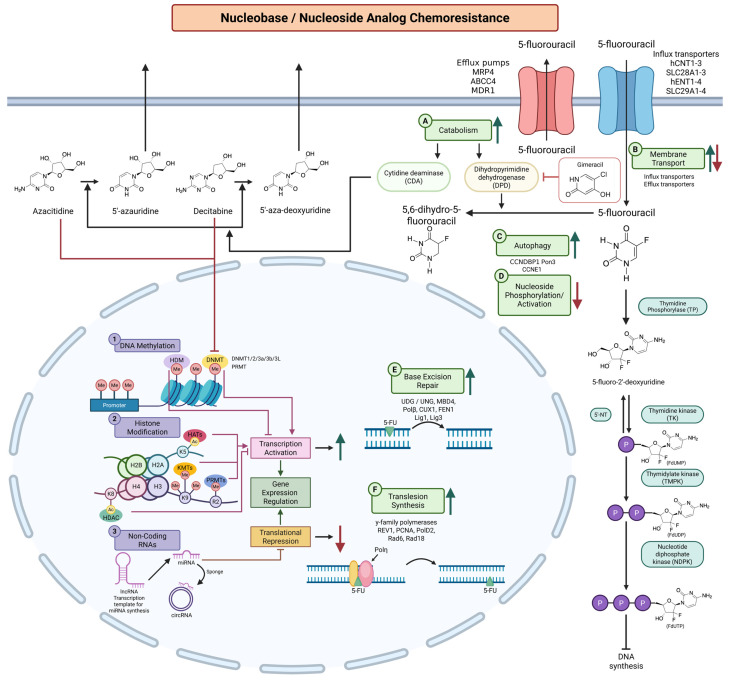
Epigenetic and molecular mechanisms contributing to nucleobase and nucleoside analog chemoresistance. Overview of intercellular pathways modulating resistance to cytotoxic and epigenetic nucleoside analogs. (A) Catabolic enzymes, including cytidine deaminase (*CDA*) and dihydropyrimidine dehydrogenase (DPD), promote drug inactivation. (B) Membrane transport dictates drug uptake via influx transporters and efflux pumps reduce intracellular drug accumulation. (C) Autophagy activation supports cell survival under analog-induced metabolic stress. (D) Impaired nucleoside analog phosphorylation activity further decreases active nucleoside analog triphosphate incorporation into DNA. (E and F) Enhanced DNA repair pathways facilitate tolerance of analog-induced DNA damage. Epigenetic mechanisms modulate therapeutic response through DNA methylation, histone modifications, and ncRNAs. Azactidine and decitabine inhibit DNMT activity, leading to hypomethylation and transcription reactivation of tumor suppressor genes. Green arrows indicate upregulation. Red arrows indicate downregulation. Blunt-ended lines denote inhibition. The figure was adapted from Kaszycki & Kim 2025 [11], which is published under a Creative Commons Attribution (CC BY 4.0) license. Created in BioRender. Kim, M. (2026) https://BioRender.com/3ed77ca (accessed on 12 March 2026).

Multifactorial and frequently overlapping activities underlie the emergence of resistance against cytotoxic nucleoside analogs (Figure 1) [13]. Reduced drug uptake through hENT1 downregulation is one of the most studied factors. In pancreatic cancer, hENT1 has been linked to gemcitabine efficacy in multiple studies [20,21]. However, in larger clinical trials, hENT1 alone has proven to be a poor biomarker unless considered together with other regulatory processes, such as promoter methylation or histone modifications [22]. Similarly, altered drug metabolism is also one of the main reasons for resistance. Lack of deoxycytidine kinase, the enzyme required for nucleoside phosphorylation, and overexpression of cytidine deaminase (*CDA*) cause more rapid elimination of analog drugs and decrease effective intracellular concentration of the active metabolites [23]. Cancer cells can be resistant to nucleoside analog-induced DNA damage through the upregulation of the repair of DNA damage, such as the expression of translesion synthesis polymerases [24,25].

In addition to intrinsic cellular mechanisms, the tumor microenvironment represents a key extrinsic factor influencing drug response [26]. Association with the stroma, gene expression changes under the condition of hypoxia, and autophagy initiation create an immunoprotective niche that shields cancer cells from drug-induced stress [27]. These changes within the microenvironment are pronounced in solid tumors such as pancreatic cancer, where the stroma is very dense and the condition of hypoxia dominates. Therefore, resistance to cytotoxic nucleoside analogs is the outcome of an interaction between intracellular (enzymes, DNA repair) and extracellular factors within the tumor microenvironment [27].

### 2.2. Epigenetic Nucleoside Analogs

Unlike the classical cytotoxic analogs, the epigenetic nucleoside analogs, azacitidine and decitabine, are not formulated to kill cancer cells directly through the damage of DNA but rather alter the epigenetic setting of the cancer cells [28]. Decitabine is incorporated into DNA but not into RNA, while azacitidine is incorporated into DNA and RNA [3]. In DNA, the analogs covalently bind to DNA methyltransferases (DNMTs), such as DNMT1 and DNMT3A/B, as they attempt to methylate the analog [3]. The irreversible inhibition of DNMTs via such a mechanism prevents the replication process from maintaining cytosine methylation, and hence, genome-wide passive demethylation occurs. The functional consequence is the re-expression of the tumor suppressor genes that were silenced, reversal of differentiation, and reversal of oncogenic epigenetic states [3].

Although DNMTis are promising therapeutically, resistance has become a significant clinical problem in myelodysplastic syndromes (MDS) and acute myeloid leukemia (AML), where azacitidine and decitabine are the only approved agents [3]. Numerous resistance mechanisms are known, most of which are epigenetic reprogramming, with only a few involving genetic mutation. Transporter dysregulations, including hENT1 downregulation, or a decrease in drug availability due to metabolic reprogramming, i.e., inactivation of dCK or overexpression of *CDA*, all serve to limit the drug pool [29].

There is a particularly insidious form of resistance: epigenetic adaptation itself. Inhibition of DNMTs and the decrease in DNA methylation do not completely halt gene silencing, as cancer cells can strengthen alternative methods [30]. A repressive histone mark, including Histone H3 Lysine 27 trimethylation (H3K27me3) catalyzed by polycomb repressive complexes, or altered chromatin remodeling signaling pathways, can maintain a steady state that inhibits expression of key tumor suppressor genes even in the presence of hypomethylating processes. These compensatory mechanisms underscore the plasticity of the epigenome and explain why DNA methyltransferase inhibitors (DNMTis) may fail in some patients [31].

Such a complex interplay offers a stimulating rationale in terms of identifying epigenetic biomarkers that may predict and monitor response to DNMTis. In contrast to cytotoxic analogs, whose resistance is predominantly metabolic or repair-based, drug resistance to epigenetic analogs lies in a balance of forces between drug-mediated demethylation and chromatin reprogramming [32]. This has significant implications in biomarker discovery, where the predictive signatures of DNMTi response will require not only the baseline epigenome, but also the possibility of adaptive epigenetic remodeling in response to therapeutic pressure [33].

## 3. Epigenetic Regulation of Nucleoside Analog Response

Epigenetic mechanisms, specifically DNA methylation, histone modifications, and chromatin remodeling, are key to how cancer cells respond to nucleoside analog therapy (Table 1) [34]. Epigenetic marks are dynamic and reversible, unlike genetic mutation, which is permanent. It is this plasticity that provides them with significant control over drug response, but also makes them poor predictive biomarkers, as tumors may reprogram their epigenome in response to therapeutic pressure. Significantly, these epigenetic mechanisms not only mediate resistance but also provide possible therapeutic targets. Therefore, it is important to identify biomarkers that can capture both the pre-existing state and the adaptive changes occurring during therapy [35].

### 3.1. DNA Methylation

One of the most well-known epigenetic changes predicting response to nucleoside analogs is DNA methylation [59,60]. Key genes in these pathways (*SLC29A1* and *DCK*) are hypermethylated in the promoter region, resulting in transcriptional silencing and the subsequent decrease in drug uptake and activation [59]. Findings across cohorts are inconsistent: while some show strong correlations between promoter methylation and outcomes, others report weak or no association, indicating that promoter methylation is not a universal biomarker [60]. This discrepancy can be attributed to tumor heterogeneity, variations in test procedures or feedback mechanisms entailing histone-mediated repression. Global hypomethylation has also been associated with resistance owing to genomic instability and upregulation of oncogenes [61]. As an example, Long Interspersed Nuclear Element-1 (LINE-1) hypomethylation resulting from hypomethylation of repetitive elements has been posited as a predictor of relapse for the treatment of AML and MDS [62]. However, the predictive value thus far is also condition-dependent: in certain AML populations, LINE-1 hypomethylation following exposure to DNMTis was associated with enhanced survival, whereas within solid tumors, overall hypomethylation was frequently associated with worse outcomes [63]. These conflicting results point to one key limitation of global methylation signatures: they risk being nonspecific and more involved with epigenetic noise than drug-selective effects [64,65]. Precisely because this opposing behavior limits its reliability as a universal marker, current clinical trial designs increasingly prioritize locus-specific biomarkers, which offer greater biological specificity and more consistent predictive value.

Furthermore, DNA repair genes are regulated by DNA methylation, and increased expression leads to rapid and direct resistance to nucleoside analog therapies. As an illustration, silencing of mismatch repair (MMR) or base excision repair (BER) can be regulated via the process of methylation. These alterations restrict tolerance to NA-elicited lesions, and thus, the drug sensitivity is altered [66]. The above examples depict that the drug response is regulated by DNA methylation at multiple levels: transport, activation, repair, and genomic stability. The problem, however, is to distinguish between methylation events that are causal biomarkers of resistance and those that are merely correlative or consequential.

### 3.2. Histone Modifications

Histone modifications add more complexity to epigenetic regulation of nucleotide analog response [67]. Histone acetylation, for instance, Histone H3 Lysine 9 acetylation (H3K9ac), is biased towards chromatin opening, thereby facilitating greater accessibility to the transcription factors and drug entry into DNA [68]. Elevated H3K9ac is associated with tumor suppressor gene re-expression and favorable azacitidine response in hematopoietic malignancies. Conversely, low acetylation is associated with resistance, emphasizing chromatin accessibility as a key predictor of drug efficacy [69].

Resistance is often maintained via histone methylation [70]. The repressive mark H3K27me3, set by polycomb repressive complexes, upholds transcriptional repression of tumor suppressor circuits even subsequent to DNA demethylation by DNMTis [71]. It sheds light on why not every patient responds to hypomethylation, as histone modifications possess the capability to condense chromatin and suppress gene reactivation. Importantly, nucleoside analogs themselves can induce secondary histone state changes. For example, azacitidine therapy has been related to H3K27 and H3K9 modifications changed in a way that can sensitize or desensitize cancer cells, relying on the chromatin environment [72]. It proves a paradox applicable to the adaptive interplay between agent action and chromatin state, wherein histone modifications are capable of augmenting or abolishing therapeutic effects [73].

From a translational perspective, histone marks present opportunities for rational combination therapies. Preclinical evidence is available for synergies of histone deacetylase inhibitors (HDACis) with DNMTis or Enhancer of Zeste Homolog 2 (EZH2) inhibitors, inhibiting H3K27 methylation [74]. Histone-focused combos of this type may bypass compensatory resistances to nucleoside analogs. These remain experimental therapies to date, with few early-phase clinical studies having taken place, a testament to the pressing need for further validation [75].

### 3.3. Chromatin Remodeling

Remodeling complexes and nucleosome positioning, which regulate the higher-order structure of chromatin, have important effects on drug response and mutations in Switch/Sucrose Non-Fermentable (SWI/SNF) complex proteins. For instance, AT-rich Interaction Domain 1a (*ARID1A*) has the ability to alter chromatin accessibility and reduce repair efficiency on drug inclusion, thereby impacting nucleoside analog sensitivity [76]. Notably, the impact of SWI/SNF mutations is type-specific for cancers: in some tumors, loss of *ARID1A* enhances sensitivity to DNMTis, whereas in others it triggers resistance through the preservation of repressive chromatin formations. Such variability indicates the dependency of chromatin remodeling on a specific background and makes the identification of universal biomarkers difficult [77].

Apart from mutations, dynamic remodeling of nucleosomes also generates replication stress induced by nucleoside analogs [78]. Repressive nucleosome structures are not reliably eliminated through inhibitors of DNMT, and such structures may persist at tumor suppressor genes [79]. Such a conclusion indicates the epigenetic network’s redundancy and flexibility, in which different layers of regulations are able to interchangeably substitute one another in maintaining malignant phenotypes. As a solution to such complexity, newer platforms today superimpose chromatin accessibility assays, such as Assay for Transposase-Accessible Chromatin using sequencing (ATAC-seq), upon DNA methylation and histone profiles [80]. Such multi-dimensional approaches provide a more comprehensive representation of the effect of chromatin remodeling on drug responses and can contribute toward the identification of more robust predictive biomarkers.

### 3.4. Crosstalk Between Epigenetic Mechanisms

DNA methylation, histone modifications, and chromatin remodeling are usually not found in isolation. Rather, they conduct crosstalk on a massive scale that increases resistance phenotypes [81]. Promoter methylation often leads to the recruitment of histone methyltransferases and further entrenchment of repression by the deposition of H3K27me3 [82]. Equally, ncRNAs are able to recruit chromatin remodelers to specific loci, and guide nucleosome repositioning and stabilize transcriptional silencing [83]. A patient with a tumor that has hENT1 promoter hypermethylation may or may not have a poor response to gemcitabine, depending on the status of histone marks or chromatin remodeling at the same locus. Such models should be validated in large patient cohorts and tested to ensure they are reproducible since early efforts at multi-omic signatures have demonstrated success in preclinical models but have not translated successfully into clinical practice yet [22,84].

## 4. Epigenetic Biomarkers with Predictive Potential

Epigenetic modifications are not only prime drivers of resistance to nucleoside analogs but also hold great promise as predictive biomarkers for patient stratification into responders and non-responders [85]. In precision oncology, such biomarkers are of particular importance because they enable the tailoring of the tumor biology and, therefore, superior efficacy and limiting unnecessary toxicities. However, epigenetic biomarkers for the clinic are still lacking because the results among studies are inconsistent, the methods for their detection are non-uniform, and because of the plasticity of the epigenetic states themselves. This section critically reviews the most salient categories of epigenetic biomarkers, i.e., DNA methylation, histone modifications, and ncRNAs, and addresses their strengths and weaknesses as well as the new multi-omic integration strategies.

### 4.1. DNA Methylation Biomarkers

DNA methylation is one of the most extensively studied nucleoside analog response biomarkers. The best-known example is *hENT1* promoter methylation [86]. Although *hENT1* methylation holds promise for prediction within individual clinical populations, results are not consistent among studies. Hence, the status of *hENT1* should never be considered in isolation but within the overall molecular and epigenetic context [2,3]. The global methylation patterns, such as LINE-1 hypomethylation and methylation of apoptosis-related genes (e.g., *p15INK4b, DAPK1*), have also been analyzed. Though promising associations with treatment failure in AML/MDS and variable responses to azacitidine have been described, global signatures are generally not specific [87]. Hypomethylation generally reflects genomic instability rather than drug-specific effects, and it is unclear whether the putative changes in drug resistance are causally related. In this regard, although the potential of DNA methylation biomarkers has shown promising results, they are not highly predictive, and their clinical application requires validation across diverse cohorts using standardized methods.

### 4.2. Histone Modification Biomarkers

Another potential class of more predictive biomarkers is histone modifications, which are relatively underexplored. Of these, the repressive marker Histone H3 Lysine 27 trimethylation (H3K27me3) has been repeatedly identified across numerous cancers, both in solid and hematologic malignancies, as being associated with poor prognosis. H3K27me3-repressed states remain after demethylation of DNA, indicating a compensatory effect of histone marks in sustaining gene silencing. In contrast, high H3K9ac reflects an open chromatin structure that permits transcriptional activation of tumor suppressor genes in response to DNMTis [88].

High H3K9ac reflects an open chromatin structure that facilitates transcriptional activation of tumor suppressor genes in response to DNMTis. Histone-based biomarkers have clinically relevant problems. In contrast to DNA methylation, which can be measured by relatively robust assays, measurements of histone modifications are less reliable, context-specific, and more challenging to quantify in clinical specimens. Moreover, agreement is lacking on the value thresholds of high or low levels of acetylation or methylation, which also complicates their applicability [89].

However, histone marks are particularly attractive because they provide for rational combination therapy targets. As an example, DNMTis were seen to combine with HDACis or with Enhancer of Zeste Homolog 2 (EZH2) inhibitors (of H3K27me3 deposition) in preclinical models, and phase I clinical studies are underway for the same. In such a manner, histone modifications can be both a predictor and a target for therapy, and this is a dual advantage for the prediction and augmentation of nucleoside analog therapy [90].

### 4.3. Non-Coding RNAs with Epigenetic Regulatory Roles

Non-coding RNAs (ncRNAs), including microRNAs (miRNAs) and long non-coding RNAs (lncRNAs), are the latest focus of studies concerning epigenetic biomarkers. They control cellular responses to nucleoside analogs, including the regulation of DNMTs, chromatin modifier genes, and drug metabolism [91]. The miR-29 family is one of the most studied because they repress the expression of the DNMT3A and DNMT3B and render AML cells sensitive to decitabine. Clinical trials have concluded that increased baseline expression of miR-29 correlates with superior responses to DNMTis and thus could serve as a predictive marker [92]. In contrast, miR-21 upregulation has been consistently implicated in resistance to gemcitabine, primarily as an inhibition of apoptosis-related molecules like Phosphatase and Tensin Homolog (PTEN) and B-cell lymphoma 2 (Bcl-2). Regardless, miR-21 is pleiotropic and involved in numerous other oncogenic processes apart from drug resistance, and such an involvement creates an issue regarding the specificity as a marker.

There is also a role of lncRNAs in resistance. For example, HOX Transcript Antisense Intergenic RNA (*HOTAIR*) recruits the polycomb repressive complex 2 (PRC2) to silence tumor suppressor genes, thereby maintaining resistance even in the presence of DNMTis. Similarly, Long Intergenic Non-Protein Coding RNA 319 (*LINC00319*) is implicated in the regulation of chromatin structure and the development of gemcitabine resistance in pancreatic cancer [93,94].

### 4.4. Multi-Omic Integration

Single epigenetic biomarkers, although appealing, are not always robust, as epigenetic modulation is redundant and dynamic. As such, multi-omic integration, or the combination of DNA methylation, histone modifications, transcriptomic data, and ncRNA profiles, has become a more appealing approach to predictive modeling. For example, the combination of hENT1 promoter methylation, histone signatures (e.g., H3K27me3), and miRNA profiles into integrated epigenetic scores has been shown to have better predictive accuracy than single markers. In a parallel development, multi-layered machine learning models have been trained on multi-omic data and demonstrated the capacity to make more accurate predictions of DNMTi response in AML than single-layer models [95,96].

## 5. Detection Technologies for Epigenetic Biomarkers

The validation and development of epigenetic biomarkers to predict nucleoside analog response and resistance have been central to the advancement of robust and reproducible detection technologies. The modulatory effects of these agents can be largely associated with DNA methylation, histone patterns, and ncRNA influences, and as such, the assays that can detect these changes need high resolution and clinical pertinence [97]. Notably, the method of detection also influences biomarker discovery, but more importantly, determines the ability to use biomarkers in a routine clinical setting where cost, reproducibility, and scalability are of concern.

### 5.1. DNA Methylation Profiling

**Figure 2:**
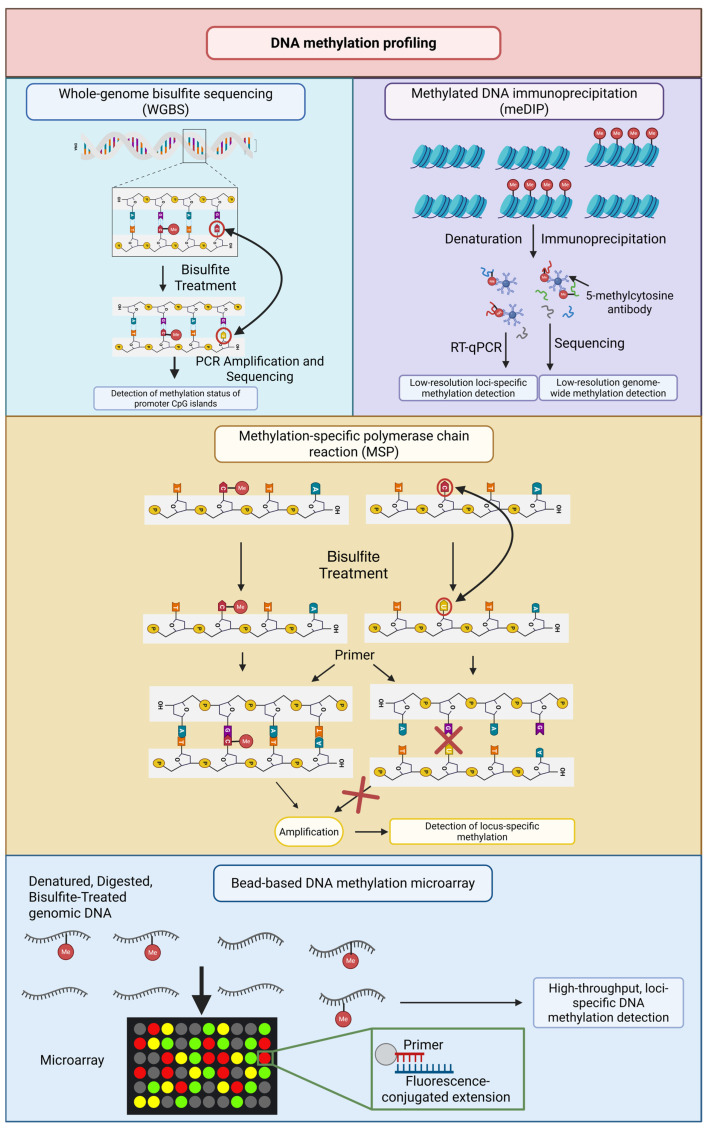
Available techniques for DNA methylation profiling. In whole-genome bisulfite sequencing (WGBS) genomic DNA is treated with bisulfite, converting unmethylated cytosines to uracils while leaving methylated cytosines unchanged. In methylated DNA immunoprecipitation (meDIP) genomic DNA is denatured and immunoprecipitated using 5-methylcytosine-specific antibodies. In methylation-specific PCR (MSP), bisulfite-treated DNA is amplified with primers specific to methylated or unmethylated sequences. In bead-based DNA methylation microarray DNA is denatured, digested, treated with bisulfite and hybridized to a microarray. Abbreviation Me stands for methyl group. Created in BioRender. Kim, M. (2026) https://BioRender.com/xhlus58 (accessed on 12 March 2026).

DNA methylation is by far the best-characterized group of biomarkers, especially in the case of DNMTis. There are several technologies in use today with various strengths and weaknesses (Figure 2). The gold standard is whole-genome bisulfite sequencing (WGBS), which can provide single-base resolution of the entire genome. Nonetheless, it is too expensive and resource-demanding to be used in large-scale clinical settings. Reduced Representation Bisulfite Sequencing (RRBS) provides a more economical option, as it preferentially targets Cytosine-phosphate-Guanine (CpG)-rich regions, potentially missing loci of interest outside of CpG islands [98].

Methylation-specific polymerase chain reaction (MSP) has been used in clinical biomarker studies because it is sensitive and locus-specific [99]. Many genes have been investigated using this technique, including Cyclin-dependent kinase 4 inhibitor B (*CDKN2B*) and Methylguanine-DNA Methyltransferase (*MGMT*). However, the nature of MSP limits its potential, as it is not designed to characterize the overall methylation pattern needed to determine multifactorial drug resistance. There has been a trade-off between cost and genome coverage with DNA methylation arrays, such as the Illumina Infinium Methylation EPIC platform, providing cost-effective and genome-wide coverage of more than 850,000 CpG sites [100]. These arrays are highly popular in biomarker discovery studies but are limited by reproducibility across laboratories and are confined to predetermined probe designs that may not cover novel resistance-associated loci [101].

Although the technology for profiling the methylation status is fairly advanced, clinical usefulness is precluded by insufficient interpretability and reproducibility. Site-specific and genome-wide methylation alterations are more correlated with a liability for genomic instability than with being generally translatable across cancers. Therefore, standardization of the panels for locus-specific accuracy and for extensive coverage is needed for their clinical application [99,101].

### 5.2. Histone Modification Detection

**Figure 3:**
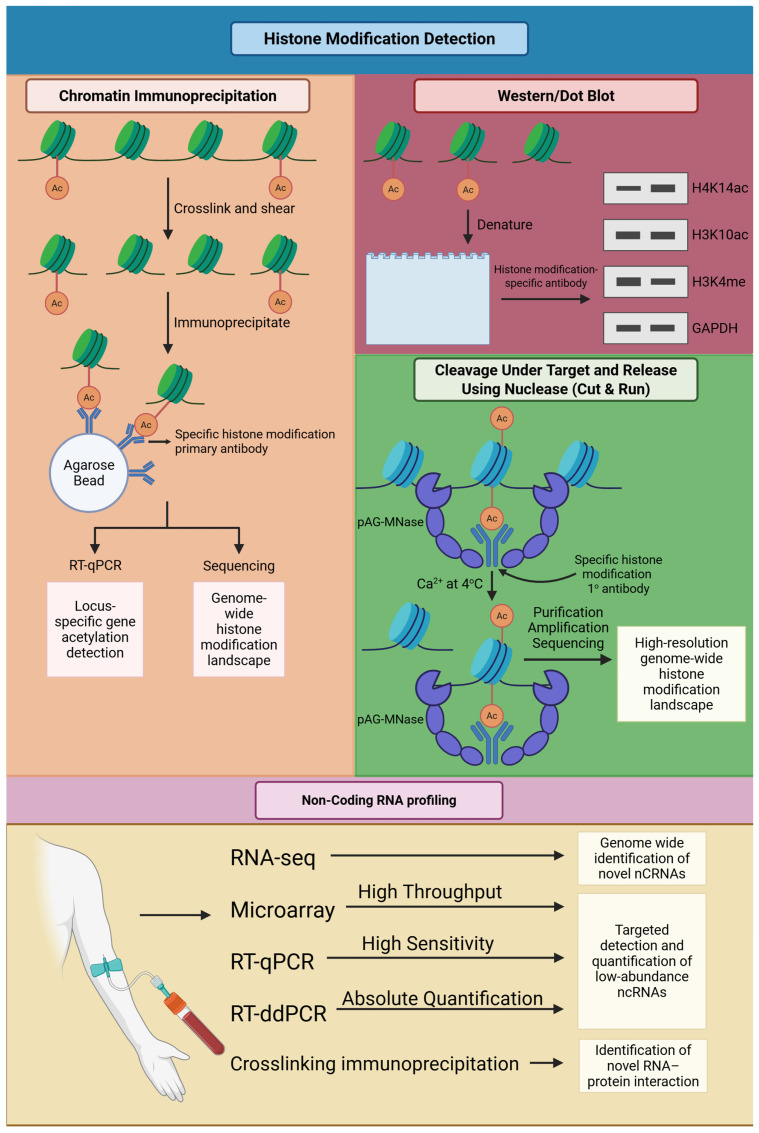
Available techniques for histone modification detection and ncRNA profiling. Histone modification can be assessed using chromatin immunoprecipitation, Western/dot blot, and Cleavage Under Targets and Release Using Nuclease (CUT and RUN). Non-coding RNA profiling methods include RNA sequencing (RNA-seq), microarrays, reverse transcription quantitative polymerase chain reaction (RT-qPCR), Reverse Transcription Droplet Digital Polymerase Chain Reaction (RT-ddPCR), and crosslinking immunoprecipitation (CLIP). Created in BioRender. Kim, M. (2026) https://BioRender.com/fy6rnu2 (accessed on 12 March 2026).

In contrast with DNA methylation, histone modifications are context-dependent and unstable, but they are informative for chromatin accessibility and transcriptional potential (Figure 3) [102,103]. Chromatin immunoprecipitation followed by sequencing (ChIP-seq) remains the gold standard for histone modification, such as H3K9me3 and H3K27me3 profiling [104]. It generates genome-wide chromatin state maps and can explain why a subset of promoters is resistant to demethylation in response to DNMTis. ChIP-seq, however, requires a lot of input material and is drastically background-noisy, making it impractical in a clinical setting [105].

To overcome such limitations, innovative technologies such as Cleavage Under Targets and Release Using Nuclease (CUT&RUN) and Cleavage Under Targets and Tagmentation (CUT&Tag) were developed [106]. They use much less input DNA, reduced background noise, and increased resolution, and are particularly suitable for sparse material with clinical specimens, such as bone marrow biopsies. While such advantages are available, usage continues to be limited to the research laboratory, as no standardized diagnostic protocol for the clinic exists.

The biggest challenge in identifying histone biomarkers is limited reproducibility and quantification [107]. Histone marks are dynamic and microenvironmentally responsive and therefore it is challenging to establish stable threshold values for clinical prediction purposes in comparison to DNA methylation [108]. Additional standardization of protocols, as well as compatibility with corresponding assays such as DNA methylation profiling or RNA-seq, may boost the strength of histone biomarker assays [105].

### 5.3. Non-Coding RNA Profiling

Non-coding RNAs (ncRNAs), like microRNAs (miRNAs) and long non-coding RNAs (lncRNAs), are promising for the development of biomarkers (Figure 3). RNA sequencing (RNA-seq) can yield a total transcriptome profile and can therefore determine resistance-related signatures for coding and non-coding genes [109]. RNA-seq is, however, extremely resource-intensive and can only be analyzed through advanced bioinformatic tools, a consideration currently limiting it from wide application in routine clinics. Microarrays provide a more specific and cost-effective alternative, particularly for profiling of known resistance-related miRNAs. As an example, the miR-29 family suppresses the expression of DNMT3A/3B, and miR-21 regulates the apoptotic process. However, microarrays are constrained by predetermined probes and are therefore not amenable for the identification of new ncRNAs [110,111].

The most universally accepted method in the validation of candidate ncRNA biomarkers is quantitative reverse transcription PCR (qRT-PCR) because of its sensitivity and clinical feasibility [112]. Significantly, circulating ncRNAs can be identified in liquid biopsies, which offer a non-invasive outcome of treatment. However, one of the main weaknesses is the absence of tumor specificity because circulating ncRNAs could be produced by several tissues, and hence, false positives are possible. ncRNAs are promising biomarkers for non-invasive monitoring but they have not been adopted due to their variability and absence of standardization across studies [113,114]. Before they can be applied in routine practice, they should be developed as standardized panels and rigorously validated across cohorts.

### 5.4. Single-Cell and Multi-Omics Approaches

Tumor heterogeneity is a key factor in resistance, and bulk profiling tends to obscure the role of small resistant subpopulations [115]. Recent advances in single-cell epigenomics have allowed us to address clonal heterogeneity at scale. Single-cell ATAC-seq (Assay for Transposase-Accessible Chromatin using sequencing), single-cell RNA-seq, and single-cell methylome sequencing will be used to identify resistant subpopulations by mapping chromatin accessibility, gene expression, and DNA methylation patterns at the single-cell level [116]. Such strategies have already revealed epigenetically heterogeneous clones in AML that are resistant to DNMTi therapy, providing mechanistic insight into relapse. Meanwhile, multi-omics (epigenomic, transcriptomic, and proteomic data) will give a system-level picture of how epigenetic perturbations are engineering a response to drugs [117]. The multi-omic data have been integrated to train machine learning models that have been reported to be more accurate in their predictions than single-omic biomarkers. However, the methods are not highly translational because of their technicalities, cost, and the need for computing infrastructure, which is not a standard practice in clinical practice. The most developed methods in the biomarker discovery area are single-cell and multi-omic methods, but they have limited direct clinical utility. The key to translating research into practice is simplification of workflows, cost-cutting, and standardization of data integration pipelines.

### 5.5. Clinical Implementation and Challenges

Recent advances in single-cell epigenomics have made it possible to solve clonal heterogeneity at scale. Although there have been massive advancements in the technological sector, epigenetic detection of biomarkers in the clinical setup is still in its infancy due to several challenges. Its primary limitation is that there is a limited amount of input material available in clinical specimens, such as bone marrow aspirates and biopsies, which makes high-resolution methods such as WGBS or ChIP-seq impractical [118]. Additionally, standardization across platforms presents difficulties, as the outcomes of sequencing and array-based technologies are not always comparable, which complicates biomarker validation. Moreover, it is also an important question to distinguish between causal and passenger epigenetic changes that directly affect drug response. The consequence of this is that in the absence of functional validation, a large proportion of candidate biomarkers turn out to be false leads. Another challenge is integrating epigenetic biomarkers with functional evaluation and clinical outcomes. This is illustrated by the fact that, although global methylation patterns can be correlated with response to azacitidine, they are not necessarily predictive when controlled by genetic mutation or cytogenetic risk factors [119]. Similarly, ncRNA signatures often lack reproducibility across different patient cohorts when used as standalone predictors, limiting their broader clinical applicability.

## 6. Clinical Evidence of Epigenetic Biomarkers in Nucleoside Analog Response

The translation of epigenetic biomarkers into predictive tools that would be utilized to determine the response to nucleoside analog therapies has become an area of great interest in current-day research. This is of specific interest in hematologic malignancies such as MDS and AML, where DNMTis such as azacitidine and decitabine are standard of care [110]. Unlike traditional chemotherapeutic agents, which affect the epigenome by damaging DNA, DNMTis affect the epigenome by changing it directly, and there is an urgent need for biomarkers that can be used to identify responsive patient populations and guide treatments [120]. Although the findings of most studies are encouraging, the field remains beset by inconsistent findings, research design heterogeneity, and a lack of coherent evidence [121].

### 6.1. DNA Methylation Profiles

Several clinical studies confirm the predictive role of DNA methylation profiles in nucleoside analog response, although not without inconsistencies [122]. Overall survival and response rates have been found to correlate with LINE-1-measured global hypomethylation in patients with MDS and AML treated with DNMTis [123]. Other studies, however, have observed that LINE-1 hypomethylation is not drug-specific but is related to general genomic instability, which casts doubt on its specificity as a predictive marker [124]. These discrepancies underscore the difficulty of distinguishing causal methylation changes from passenger changes within clinical specimens and imply the possibility that the benefit from therapy might be contingent on re-expression of repressed cell cycle regulators. Nevertheless, heterogeneity among responses to therapy is not describable solely through methylation signatures because patients with hypermethylation are not consistently sensitive to therapy [125]. More recently, increased sensitivity to DNMTis has been linked with basal immune-related genes, for example, Cytotoxic T-Lymphocyte Antigen 4 (*CTLA4*) and Programmed Death-Ligand 1 (*CD274*), and an immunomodulatory mechanism is indicated as a second-line therapeutic action [126]. These findings are promising, but the questions about replicability in genetically diverse populations remain unaddressed.

### 6.2. DNMT Expression Levels

Besides methylation status, the expression of DNMTs themselves has also been studied as a proteomic biomarker. The increase in DNMT1 and DNMT3A expression is always associated with DNMTi resistance [127]. Clinical data shows that patients with low baseline DNMT1 are more likely to respond to azacitidine and, therefore, therapeutic responses can be preconditioned by the inherent dependency on DNMT [128]. These are promising results, but not conclusive; the overexpression of DNMT is not always independent from other oncogenic drivers, and, therefore, it is difficult to determine its independent prognostic relevance [129]. Also, the expression of DNMT is not always stable and may vary during treatment; therefore, it is not a good biomarker of stability [130].

### 6.3. Histone Modification Signatures

Although DNMTis have shown significance in nucleoside analog response, histone modification levels have also been found to be important determinants of durable responses [131]. Worsening of outcomes has been associated with increased repressive marks such as H3K9me3 and H3K27me3, which impede reactivation of transcription despite the demethylation of the DNA [132]. This resistance explains why some patients fail to respond to treatment, even when DNA demethylation occurs extensively, because compensatory mechanisms such as repressive histone marks or chromatin remodeling maintain gene silencing [133]. However, histone biomarkers have not been used widely in the clinic, as there has been a technical issue in uniform detecting histone modifications in patient biopsies together with inconsistency across cancer types [5].

### 6.4. MicroRNAs (miRNAs) as Biomarkers

Circulating and tumor-specific microRNAs (miRNAs) present an appealing biomarker development opportunity, due to their stability in biofluids and the possibility of non-invasive monitoring [134]. The most consistently studied is the miR-29 family, with high baseline expression correlating with better decitabine response in AML patients. This observation is consistent mechanistically with the capacity of miR-29 to inhibit DNMT3A and DNMT3B, thus making cells sensitive to DNMT inhibition [135]. Notably, miRNA-based signatures are also being investigated as early indicators of relapse, which would be of clinical value in monitoring patients [136]. Nevertheless, the specificity of circulating miRNAs is a matter of concern since they may be produced by both tumor and non-tumor tissues, which may result in false positives [137].

### 6.5. Epigenetic Biomarkers in Solid Tumors

It is important to recognize that the application of epigenetic biomarkers and therapies in solid tumors presents distinct challenges. Unlike in blood cancers, solid tumors are characterized by a complex tumor microenvironment, greater cellular heterogeneity, and altered drug penetration. DNA methylation and histone modification patterns remain clinically informative across both cancer types; however, their predictive utility may be diminished in solid tumors due to additional biological barriers. Although testing and analysis for solid tumors is more difficult, blood-based screening technology is improving. For example, Guardant SHIELD is a qualitative blood-based diagnostic for colorectal cancer screening that was FDA-approved in July 2024, which detects specific somatic mutations and fragmentation in cfDNA (cell-free DNA). Recent improvements have incorporated DNA methylation pattern analysis to allow for increased tumor-specific determination and increased sensitivity [138]. Histone modifications can also be detected through cfDNA through analysis of the fragmentation, which can provide information on nucleosome positioning and chromatin accessibility, therefore providing diagnostic information on gene expression, tissue of origin, and tumor vs. normal chromatin states [139]. MicroRNAs have been of great interest to researchers because of their relatively high stability in blood and their tumor-specific expression patterns allowing for their detection and therapy response prediction of multiple solid tumors such as colorectal, lung, and breast cancer [140].

### 6.6. Integration with Clinical Outcomes

Epigenetic biomarker studies are being applied in large clinical trials to better select patients. In the AZA-001 trial for higher-risk MDS, epigenetic alteration, especially DNA hypomethylation, was linked to better overall survival of patients receiving azacitidine [3]. Similarly, the DECIDER trial included methylation profiling to identify the patients with AML who would respond optimally to decitabine [141]. These trials demonstrate the proof-of-concept that epigenetic biomarkers can be used to predict treatment outcomes (Table 2). Most recently, current trials are expanding this paradigm to solid tumors, testing whether baseline epigenetic states can predict nucleoside analog efficacy across various malignancies [142]. However, the translation into practice is still low: correlations are strong in retrospective studies, but prospective biomarker-based trial designs are rare [143,144].

### 6.7. Challenges in Clinical Translation

Although there is promising evidence, there are still some major obstacles that do not allow for the use of epigenetic biomarkers on a large scale in clinical practice. There is also technical variability between detection platforms to add to the problem: results vary dramatically when methylation is detected by bisulfite sequencing, arrays, or targeted PCR-based analyses. Lastly, most of the clinical studies are either too small or retrospective in nature; thus, it is hard to determine the causality between biomarker status and response to treatment [149].

The available evidence suggests that epigenetic biomarkers are promising, but their clinical application remains largely exploratory. Without standardization, promising isolated evidence can plateau at the preclinical level and prevent the clinical translation of epigenetic biomarkers of nucleoside analog therapy [150]. To facilitate the clinical translation of these biomarkers, it is important to consider the practical realities of routine workflows. Successful implementation will require assays that provide rapid and reliable results, are compatible with point-of-care laboratory settings, and adhere to standardized, Clinical Laboratory Improvement Amendments-certified protocols. Developing such platforms is critical to ensure that promising biomarkers can be effectively integrated into clinical decision-making and clinical trial designs.

## 7. Future Directions

Research on epigenetic biomarkers and response to nucleoside analogs is advancing rapidly; however, practical use in the clinic remains a challenge. Heterogeneity of the epigenetic profiles, both within an individual tumor and across patients, is one of the biggest issues [151]. Patterns of DNA methylation and histone modifications are not only distinct for various cancers but are also distinct from one tumor clone in a patient to another [62]. Biomarkers predictive in one type of cancer are not in another, raising issues regarding their reliability in a clinical environment [121]. Technical variability also limits reproducibility. Sample collection, processing, and analytic pipeline variability routinely produce inconsistent results between labs [99,101]. While whole-genome bisulfite sequencing offers unparalleled resolution, it is too costly for routine use [152]. Targeted assays and methylation arrays are more practical, but not always sufficiently sensitive to offer a global view of the complexity in mechanism of resistance. Standardization of platforms and workflows is therefore needed to enable safe transition of epigenetic biomarkers.

Another challenge is how to integrate epigenetic biomarkers with additional molecular layers. Drug responses are seldom determined on the basis of epigenetics only; gene mutations, remodeling of the transcriptome, and protein adaptations commonly co-exert themselves in the production of therapeutic effects. Multi-omics platforms are capable of generating a system-level picture of the biology of drug resistance, and machine learning might assist in assembling such complex data sets in the construction of prediction models. Nonetheless, they are not mature yet in clinical oncology [95,96].

Resistance also occurs through compensation mechanisms. Despite effective DNMT inhibition, tumors are capable of evading therapy through histone-dependent repression, chromatin remodeling, or various metabolic processes, including (Ten-Eleven Translocation) TET mutations [62]. These mechanisms emphasize rational combination therapies. Preclinical models establish that rational therapies through the use of a combination of DNMTis and HDACis, immune checkpoints inhibitors, or EZH2 inhibitors are capable of overcoming resistance and achieving durability of therapy [8,9]. Translation of such methods to the clinic will require validated panels of biomarkers for identifying patients who are maximally likely to benefit.

Finally, clinical trial design remains a key bottleneck. Most biomarker analyses are small, retrospective, or exploratory in design, and prospectively planned biomarker-driven studies with randomization of treatment allocation on the basis of epigenetic signature are currently extremely rare. Preparatory to such studies, wide-scale clinical practice and drug agency approval of biomarker-guided nucleoside analog therapy are therefore unlikely.

To overcome these obstacles, focus should be on the generation of standardized biomarker panels of DNA methylation patterns, histone modification profiles, and ncRNA signatures. These panels, in turn, will need to be validated in large, multi-center patient populations. Non-invasive liquid biopsy methods might offer real-time tracking of resistance, and machine learning and multi-omic integration might further boost predictive power. Eventually, rationally engineered combination regimens with insightful biomarker signatures on hand and validated in prospective clinical studies will realize the therapeutic potential of epigenetic biomarkers in nucleoside analog therapy.

## 8. Conclusions

Cytotoxic and epigenetic nucleoside analogs remain the mainstay of the practice of oncology, yet their clinical efficacy is limited by inter-patient heterogeneity and the universal emergence of resistance. Epigenetic biomarkers like DNA methylation profiles, histone modifications, and ncRNA possess great promise for the prediction of the response to therapy, the determination of the mechanism of resistance, and the establishment of precision medicine. Their clinical translation has been limited by tumor heterogeneity, variability of the assays, and the reversible nature of epigenetic states. The future of the field is the establishment of standard panels of biomarkers, real-time monitoring through liquid biopsy methods, and the integration of multi-omics data enhanced by machine learning. To achieve the predictive and therapeutic promise of epigenetic biomarkers fully, rational combination therapies must be tested in forward-phase, biomarker-guided clinical trials. Resolution of these challenges shall enable nucleoside analog therapy to become more targeted and tailored, ultimately enhancing therapeutic efficacy while minimizing resistance.

## Figures and Tables

**Table 1:** Epigenetic biomarkers associated with nucleoside analog response and resistance in cancer. Summary of ncRNAs, histone modifications, and DNA methylation-related alterations implicated in resistance to nucleoside analog drugs. Categorization of biomarkers by epigenetic regulation type, regulation direction, chromatin state, mechanism of resistance, and cancer type. The final column summarizes commonly used profiling and detection methods.

Epigenetic Biomarkers in Nucleoside Analog Response/Resistance								
Type	Regulation	Direction	Effect on Chromatin	Regulated Genes	Mechanism of Resistance	Cancer Type	Resistant Nucleoside	Profiling/Detection Methods
Non-coding RNAs	N/A			*PVT1* [36] (lncRNA)	Increased nucleobase export	Breast	Gemcitabine	RNA-seq Microarray RT-qPCR Crosslinking immunoprecipitation (CLIP/CLIP-seq)
				*GHET1* [37] (lncRNA)				
				*MTHFD1L* [38] (circRNA)	Increased DNA repair	Pancreatic		
				*CRATIG* [39] (lncRNA)	Decreased DNA damage response	Colorectal	5-fluorouracil	
				miR-274-3p [40] (miRNA)				
Histone modifications	Histone acetyltransferases (HATs)	Upregulation	Euchromatin	*CSNK2A1* [41]	Increased autophagy	Pancreatic	Gemcitabine	Chromatin immunoprecipitation sequencing/quantitative PCR (CHIP-seq/qPCR) Western/Dot blot Cleavage Under Targets and Release Using Nuclease (CUT&RUN)
				*PIK3CA* [42]				
				*TYMS* [43]	Analog metabolism	Colorectal	5-fluorouracil	
				*DPYD* [44]				
	Histone deacetylases	Downregulation	Heterochromatin	*CDKN1A* [45]	Decreased DNA damage response	Colorectal	5-fluorouracil	
				*SPHK2* [46]	Analog metabolism			
				*TGIF1* [47]	DNA damage response	Bladder	Gemcitabine	
				*H2AFX* [48]		Pancreatic		
DNA methylation	DNA methyltransferases (DNMTs) DNMT1, DNMT2, DNMT3a/b, DNMT3L, PRMT	Upregulation (Hypomethylation)	Open promoter	*ABCB1* [49]	Increased nucleoside export	Breast	Gemcitabine	Whole-genome and Reduced Representation Bisulfite Sequencing (WGBS/RRBS) Methylation-specific polymerase chain reaction (MSP) Methylated DNA immunoprecipitation (meDIP) Bead-based DNA methylation microarray
				*ABCB2* [50]				
				*MGMT* [51]	Increased DNA repair	Pancreatic		
				*NFE2L2* [52]	Relives oxidative stress	Colorectal	5-fluorouracil	
				*ABCB1* [53]	Increased nucleobase export	Gastric		
		Downregulation (Hypermethylation)	Closed promoter	*TFAP2E* [54]	Decreased DNA damage response			
				*BNIP3* [55]	Increased autophagy	Pancreatic	Gemcitabine	
				*CCNE1* [56]		Colorectal	5-fluoroiracil	
				*CCNDBP1* [57]				
				*PON3* [58]				

**Table 2:** Clinical trials involving epigenetic therapies and biomarkers in the treatment of various cancers. Summary of epigenetic therapies and biomarkers in relation to resistance to nucleoside analog drugs. Categorization of biomarkers by trail name, trail type, number of participants, cancer type, epigenetic therapy or biomarker and significant findings from the study.

Clinical Trial	Interventional or Observational	Phase	Number of Participants	Cancer Type	Epigenetic Therapy	Epigenetic Involvement	Notes
DECIDER (DRKS00000733) [141]	Interventional	Phase II	*n* = 200	Acute myeloid leukemia (AML)	Decitabine	DNA methyltransferase inhibitor	The addition of HDAC inhibitor valproic acid to decitabine results in an objective response rate of 17.6%, compared to 8.5% with decitabine alone; however, this difference was not statistically significant (*p* = 0.88)
					Valproic acid	Histone deacetylase inhibitor	
					All-trans retinoic acid		
Jensen et al., 2019 [86]	Observational (retrospective)	Not applicable	*n* = 5351	Colorectal cancer (CRC)	Not applicable	DNA methylation profiling CpG sites in cell-free DNA (blood samples) Microarray + bisulfite sequencing	Biomarkers identified: C9orf50 KCNQ5 CLIP4 Highly accurate non-invasive CRC detection using circulating tumor DNA methylation
Sun et al. [142]	Observational (preclinical)	Not applicable	*n* = 600 (cancer cell lines)	Multiple cancer types	Decitabine Azacitidine Zebularine RG108	DNA methylation (CpG site) profiling	DNA methylation strongly predicts response to decitabine with over 100,000 CpG sites
NCT01074047 [145]	Interventional	Phase III	*n* = 488	Acute myeloid leukemia	Azacitidine	DNA methyltransferase inhibition	Median overall survival improved in azacitidine compared to conventional treatment (10.4 vs. 6.5 months)
VIALE-A (NCT02993523) [146]	Interventional	Phase III	*n* = 431	Acute myeloid leukemia	Azacitidine	DNA methyltransferase inhibition	Improved overall survival with dual treatment with venetoclax compared to azacitidine alone
NCT01639131 [147]	Interventional	Phase II	*n* = 6	Metastatic colorectal cancer	Not applicable	DNA methylation profiling Checkpoint with forkhead and Ring Finger Domains (CHFR) promoter methylation biomarkers	CHFR promoter methylation predicted sensitivity to gemcitabine
(SPIRE) ISRCTN 16332228 [148]	Interventional	Phase I	*n* = 20	Multiple solid malignancies	Guadecitabine	DNA methyltransferase inhibition	Epigenetic priming enhances response to gemcitabine and cisplatin

## Data Availability

No new data were created or analyzed in this study. Data sharing is not applicable to this article.

## Abbreviations

AML—acute myeloid leukemia; ATAC-seq—Assay for Transposase-Accessible Chromatin using sequencing; BER—base excision repair; *CDA*—cytidine deaminase; cfDNA—cell-free DNA; CHFR—Checkpoint with forkhead and Ring Finger Domains; ChIP-seq—chromatin immunoprecipitation sequencing; DNMT—DNA methyltransferase; DNMTis—DNA methyltransferase inhibitors; EZH2—Enhancer of Zeste Homolog 2; HDACis—histone deacetylase inhibitors; hENT1—human Equilibrative Nucleoside Transporter 1; LINE-1—Long Interspersed Nuclear Element-1; lncRNA—long non-coding RNA; MDS—myelodysplastic syndrome; miRNA/miR—microRNA; MMR—mismatch repair; MSP—methylation-specific PCR; NA—nucleoside analog; ncRNA—non-coding RNA; PRC2—polycomb repressive complex 2; qRT-PCR—quantitative reverse transcription polymerase chain reaction; RNA-seq—RNA sequencing; RRBS—Reduced Representation Bisulfite Sequencing; SWI/SNF—Switch/Sucrose Non-Fermentable chromatin remodeling complex; WGBS—whole-genome bisulfite sequencing.

## References

[1] Baroud M., Lepeltier E., Thepot S., El-Makhour Y., Duval O. (2021). The evolution of nucleosidic analogues: Self-assembly of prodrugs into nanoparticles for cancer drug delivery. Nanoscale Adv..

[2] Ilango S., Paital B., Jayachandran P., Padma P.R., Nirmaladevi R. (2020). Epigenetic alterations in cancer. Front. Biosci. (Landmark Ed.).

[3] Bohl S.R., Bullinger L., Rücker F.G. (2018). Epigenetic therapy: Azacytidine and decitabine in acute myeloid leukemia. Expert Rev. Hematol..

[4] Bure I.V., Nemtsova M.V., Kuznetsova E.B. (2022). Histone modifications and non-coding RNAs: Mutual epigenetic regulation and role in pathogenesis. Int. J. Mol. Sci..

[5] Xu X., Peng Q., Jiang X., Tan S., Yang Y., Yang W., Han Y., Chen Y., Oyang L., Lin J. (2023). Metabolic reprogramming and epigenetic modifications in cancer. Exp. Mol. Med..

[6] Safi S. (2024). Epigenetic modulation in cancer: Molecular mechanisms and therapeutic targets. Prem. J. Sci..

[7] Yehia H. (2025). Precision medicine engaging nucleoside analogues. Adv. Tradit. Med..

[8] Garner I.M., Brown R. (2022). Is There a Role for Epigenetic Therapies in Modulating DNA Damage Repair Pathways to Enhance Chemotherapy and Overcome Drug Resistance?. Cancers.

[9] Zhan C., Tang T., Wu E., Zhang Y., He M., Wu R., Bi C., Wang J., Zhang Y., Shen B. (2023). Multi-omics approaches to personalized medicine in myocardial infarction. Front. Cardiovasc. Med..

[10] Huff S.E., Winter J.M., Dealwis C.G. (2022). Inhibitors of the Cancer Target Ribonucleotide Reductase, Past and Present. Biomolecules.

[11] Kaszycki J., Kim M. (2025). Epigenetic drivers of chemoresistance in nucleobase and nucleoside analog therapies. Biology.

[12] De Jonghe S., Herdewijn P. (2022). Overview of marketed nucleoside and nucleotide analogs. Curr. Protoc..

[13] Tsesmetzis N., Paulin C.B., Rudd S.G., Herold N. (2018). Nucleobase and Nucleoside Analogues: Resistance and Re-Sensitisation at the Level of Pharmacokinetics, Pharmacodynamics and Metabolism. Cancers.

[14] Averill J.R., Jung H. (2023). Mutagenic incorporation of inosine into DNA via T:I mismatch formation by human DNA polymerase eta. Biochem. J..

[15] Lin J.C., Averill J.R., Jung H. (2025). Deciphering the Role of DNA Polymerase Eta on the Incorporation and Bypass of Inosine and Cell Cycle Arrest. Int. J. Mol. Sci..

[16] Averill J.R., Lin J.C., Jung J., Jung H. (2024). Novel insights into the role of translesion synthesis polymerase in DNA incorporation and bypass of 5-fluorouracil in colorectal cancer. Nucleic Acids Res..

[17] Fordham S.E., Blair H.J., Elstob C.J., Plummer R., Drew Y., Curtin N.J., Heidenreich O., Pal D., Jamieson D., Park C. (2018). Inhibition of ATR acutely sensitizes acute myeloid leukemia cells to nucleoside analogs that target ribonucleotide reductase. Blood Adv..

[18] da Costa A.A.B., Chowdhury D., Shapiro G.I., D’Andrea A.D., Konstantinopoulos P.A. (2023). Targeting replication stress in cancer therapy. Nat. Rev. Drug Discov..

[19] Plunkett W., Gandhi V. (2021). Nucleoside analogs: Cellular pharmacology and mechanisms. Nucleoside Analogs in Cancer Therapy.

[20] Pastor-Anglada M., Pérez-Torras S. (2018). Emerging roles of nucleoside transporters. Front. Pharmacol..

[21] Elander N., Aughton K., Ghaneh P., Neoptolemos J., Palmer D., Cox T., Campbell F., Costello E., Halloran C., Mackey J. (2018). Expression of dihydropyrimidine dehydrogenase (DPD) and hENT1 predicts survival in pancreatic cancer. Br. J. Cancer.

[22] Xiao J., Zhao F., Luo W., Yang G., Wang Y., Qiu J., Liu Y., You L., Zheng L., Zhang T. (2024). Human equilibrative nucleoside transporter 1: Novel biomarker and prognostic indicator for patients with gemcitabine-treated pancreatic cancer. Cancer Manag. Res..

[23] Ligasová A., Piskláková B., Friedecký D., Koberna K. (2023). A new technique for the analysis of metabolic pathways of cytidine analogues and cytidine deaminase activities in cells. Sci. Rep..

[24] Saiki Y., Yoshino Y., Fujimura H., Manabe T., Kudo Y., Shimada M., Mano N., Nakano T., Lee Y., Shimizu S. (2012). DCK is frequently inactivated in acquired gemcitabine-resistant human cancer cells. Biochem. Biophys. Res. Commun..

[25] Sharma R.A., Dianov G.L. (2007). Targeting base excision repair to improve cancer therapies. Mol. Asp. Med..

[26] Khalaf K., Hana D., Chou J.T.T., Singh C., Mackiewicz A., Kaczmarek M. (2021). Aspects of the tumor microenvironment involved in immune resistance and drug resistance. Front. Immunol..

[27] Hurra I.M., Mintoo M.J., Fatima K., Kousar R., Mohiuddin T., Khan S.U. (2024). Tumor microenvironment: Multiway role in drug resistance. Drug Resistance in Cancer: Mechanisms and Strategies.

[28] Kaszycki J., Kim M. (2025). Epigenetic regulation of transcription factors involved in NLRP3 inflammasome and NF-kB signaling pathways. Front. Immunol..

[29] Amrutkar M., Gladhaug I.P. (2021). Stellate cells aid growth-permissive metabolic reprogramming and promote gemcitabine chemoresistance in pancreatic cancer. Cancers.

[30] Naghizadeh S., Mansoori B., Mohammadi A., Sakhinia E., Baradaran B. (2019). Gene silencing strategies in cancer therapy: An update for drug resistance. Curr. Med. Chem..

[31] Beacon T.H., Delcuve G.P., López C., Nardocci G., Kovalchuk I., van Wijnen A.J., Davie J. (2021). The dynamic broad epigenetic (H3K4me3, H3K27ac) domain as a mark of essential genes. Clin. Epigenet..

[32] Shlyakhtina Y., Moran K.L., Portal M.M. (2021). Genetic and non-genetic mechanisms underlying cancer evolution. Cancers.

[33] Giri A.K., Aittokallio T. (2019). DNMT inhibitors increase methylation in the cancer genome. Front. Pharmacol..

[34] Fardi M., Solali S., Hagh M.F. (2018). Epigenetic mechanisms as a new approach in cancer treatment: An updated review. Genes Dis..

[35] Thomas M.L., Marcato P. (2018). Epigenetic modifications as biomarkers of tumor development, therapy response, and recurrence across the cancer care continuum. Cancers.

[36] Traversa D., Simonetti G., Tolomeo D., Visci G., Macchia G., Ghetti M., Martinelli G., Kristensen L., Storlazzi C. (2022). Unravelling similarities and differences in the role of circular and linear *PVT1* in cancer and human disease. Br. J. Cancer.

[37] Barth D.A., Juracek J., Slaby O., Pichler M., Calin G.A. (2020). lncRNA and mechanisms of drug resistance in cancers of the genitourinary system. Cancers.

[38] Huang Y., Zhang C., Xiong J., Ren H. (2020). Emerging important roles of circRNAs in human cancer and other diseases. Genes Dis..

[39] Wang J., Zhang X., Zhang J., Chen S., Zhu J., Wang X. (2021). Long noncoding RNA CRART16 confers 5-FU resistance in colorectal cancer cells by sponging miR-193b-5p. Cancer Cell Int..

[40] Que K., Tong Y., Que G., Li L., Lin H., Huang S., Wang R., Tang L. (2017). Downregulation of miR-874-3p promotes chemotherapeutic resistance in colorectal cancer via inactivation of the Hippo signaling pathway. Oncol. Rep..

[41] Liu Z.-D., Shi Y.-H., Xu Q.-C., Zhao G.-Y., Zhu Y.-Q., Li F.-X., Ma M.-J., Ye J.-Y., Huang X.-T., Wang X.-Y. (2024). CSNK2A1 confers gemcitabine resistance to pancreatic ductal adenocarcinoma via inducing autophagy. Cancer Lett..

[42] Liu K., Geng Y., Wang L., Xu H., Zou M., Li Y., Zhao Z., Chen T., Xu F., Sun L. (2022). Systematic exploration of the underlying mechanism of gemcitabine resistance in pancreatic adenocarcinoma. Mol. Oncol..

[43] Kumar A., Singh A.K., Singh H., Thareja S., Kumar P. (2022). Regulation of thymidylate synthase: An approach to overcome 5-FU resistance in colorectal cancer. Med. Oncol..

[44] Cui Z., He S., Wen F., Lu L., Xu L., Wu H., Wu S. (2023). Dihydropyrimidine dehydrogenase (DPD) as a bridge between the immune microenvironment of colon cancers and 5-FU resistance. Front. Biosci. (Landmark Ed.).

[45] Maiuthed A., Ninsontia C., Erlenbach-Wuensch K., Ndreshkjana B., Muenzner J.K., Caliskan A., Husayn A.P., Chaotham C., Hartmann A., Roehe A.V. (2018). Cytoplasmic p21 mediates 5-fluorouracil resistance by inhibiting pro-apoptotic Chk2. Cancers.

[46] Zhang Y.-H., Shi W.-N., Wu S.-H., Miao R.-R., Sun S.-Y., Luo D.-D., Wan S.-B., Guo Z.-K., Wang W.-Y., Yu X.-F. (2020). SphK2 confers 5-fluorouracil resistance to colorectal cancer via upregulating H3K56ac-mediated DPD expression. Oncogene.

[47] Yeh B.-W., Li W.-M., Li C.-C., Kang W.-Y., Huang C.-N., Hour T.-C., Liu Z.-M., Wu W.-J., Huang H.-S. (2016). Histone deacetylase inhibitor trichostatin A resensitizes gemcitabine-resistant urothelial carcinoma cells via suppression of TG-interacting factor. Toxicol. Appl. Pharmacol..

[48] Voutsadakis I.A. (2011). Molecular predictors of gemcitabine response in pancreatic cancer. World J. Gastrointest. Oncol..

[49] Lu Y., Xu D., Peng J., Luo Z., Chen C., Chen Y. (2019). HNF1A inhibition induces the resistance of pancreatic cancer cells to gemcitabine by targeting ABCB1. EBioMedicine.

[50] Xu M., Li L., Liu Z., Jiao Z., Xu P., Kong X., Huang H., Zhang Y. (2013). ABCB2 (TAP1) as the downstream target of SHH signaling enhances pancreatic ductal adenocarcinoma drug resistance. Cancer Lett..

[51] Shi Y., Wang Y., Qian J., Yan X., Han Y., Yao N., Ma J. (2020). MGMT expression affects the gemcitabine resistance of pancreatic cancer cells. Life Sci..

[52] Shen C.J., Lin P.L., Lin H.C., Cheng Y.W., Huang H.S., Lee H. (2019). RV-59 suppresses cytoplasmic Nrf2-mediated 5-fluorouracil resistance and tumor growth in colorectal cancer. Am. J. Cancer Res..

[53] Khakbaz P., Panahizadeh R., Vatankhah M.A., Najafzadeh N. (2021). Allicin reduces 5-fluorouracil-resistance in gastric cancer cells through modulating MDR1, DKK1, and WNT5A expression. Drug Res..

[54] Sun J., Wang X., Zha J., Li W., Li D., Xu H. (2019). *TFAP2E* methylation promotes 5-fluorouracil resistance via exosomal miR-106a-5p and miR-421 in gastric cancer MGC-803 cells. Mol. Med. Rep..

[55] Ishida M., Sunamura M., Furukawa T., Akada M., Fujimura H., Shibuya E., Egawa S., Unno M., Horii A. (2007). Elucidation of the relationship of *BNIP3* expression to gemcitabine chemosensitivity and prognosis. World J. Gastroenterol..

[56] Blondy S., David V., Verdier M., Mathonnet M., Perraud A., Christou N. (2020). 5-Fluorouracil resistance mechanisms in colorectal cancer: From classical pathways to promising processes. Cancer Sci..

[57] Huang R., Lin J.Y., Chi Y.J. (2018). MiR-519d reduces the 5-fluorouracil resistance in colorectal cancer cells by down-regulating the expression of CCND1. Eur. Rev. Med. Pharmacol. Sci..

[58] Baharudin R., Ab Mutalib N.-S., Othman S.N., Sagap I., Rose I.M., Mohd Mokhtar N., Jamal R. (2017). Identification of predictive DNA methylation biomarkers for chemotherapy response in colorectal cancer. Front. Pharmacol..

[59] Buocikova V., Tyciakova S., Pilalis E., Mastrokalou C., Urbanova M., Matuskova M., Demkova L., Medova V., Longhin E.M., Rundén-Pran E. (2022). Decitabine-induced DNA methylation-mediated transcriptomic reprogramming in human breast cancer cell lines; the impact of DCK overexpression. Front. Pharmacol..

[60] Sorrentino V.G., Thota S., Gonzalez E.A., Rameshwar P., Chang V.T., Etchegaray J.P. (2021). Hypomethylating chemotherapeutic agents as therapy for myelodysplastic syndromes and prevention of acute myeloid leukemia. Pharmaceuticals.

[61] Romero-Garcia S., Prado-Garcia H., Carlos-Reyes A. (2020). Role of DNA methylation in the resistance to therapy in solid tumors. Front. Oncol..

[62] Youn A., Kim K.I., Rabadan R., Tycko B., Shen Y., Wang S. (2018). A pan-cancer analysis of driver gene mutations, DNA methylation and gene expressions reveals that chromatin remodeling is a major mechanism inducing global changes in cancer epigenomes. BMC Med. Genom..

[63] Gezer U., Özgür E., Yörüker E.E., Polatoglou E., Holdenrieder S., Bronkhorst A. (2024). LINE-1 cfDNA methylation as an emerging biomarker in solid cancers. Cancers.

[64] Kusch N. (2021). Two-Step Models for Tumour-Drug Response Using Heterogeneous High-Dimensional Assays. Ph.D. Thesis.

[65] Michalak E.M., Burr M.L., Bannister A.J., Dawson M.A. (2019). The roles of DNA, RNA and histone methylation in ageing and cancer. Nat. Rev. Mol. Cell Biol..

[66] Sharma N., Pasala M.S., Prakash A. (2019). Mitochondrial DNA: Epigenetics and environment. Environ. Mol. Mutagen..

[67] Ueda M., Seki M. (2020). Histone modifications form epigenetic regulatory networks to regulate abiotic stress response. Plant Physiol..

[68] Chen Y., Liang R., Li Y., Jiang L., Ma D., Luo Q., Song G. (2024). Chromatin accessibility: Biological functions, molecular mechanisms and therapeutic application. Signal Transduct. Target. Ther..

[69] Ozyerli-Goknar E., Bagci-Onder T. (2021). Epigenetic deregulation of apoptosis in cancers. Cancers.

[70] Yamagishi M., Kuze Y., Kobayashi S., Nakashima M., Morishima S., Kawamata T., Makiyama J., Suzuki K., Seki M., Abe K. (2024). Mechanisms of action and resistance in histone methylation-targeted therapy. Nature.

[71] Blackledge N.P., Klose R.J. (2021). The molecular principles of gene regulation by Polycomb repressive complexes. Nat. Rev. Mol. Cell Biol..

[72] Paradise B.D., Barham W., Fernandez-Zapico M.E. (2018). Targeting epigenetic aberrations in pancreatic cancer, a new path to improve patient outcomes?. Cancers.

[73] Quagliano A., Gopalakrishnapillai A., Barwe S.P. (2020). Understanding the mechanisms by which epigenetic modifiers avert therapy resistance in cancer. Front. Oncol..

[74] Hontecillas-Prieto L., Flores-Campos R., Silver A., De Álava E., Hajji N., García-Domínguez D.J. (2020). Synergistic enhancement of cancer therapy using HDAC inhibitors: Opportunity for clinical trials. Front. Genet..

[75] Wen S., Wang J., Liu P., Li Y., Lu W., Hu Y., Liu J., He Z., Huang P. (2018). Novel combination of histone methylation modulators with therapeutic synergy against acute myeloid leukemia in vitro and in vivo. Cancer Lett..

[76] Hao F., Zhang Y., Hou J., Zhao B. (2025). Chromatin remodeling and cancer: The critical influence of the SWI/SNF complex. Epigenet. Chromatin.

[77] Li Y., Yang X., Zhu W., Xu Y., Ma J., He C., Wang F. (2022). SWI/SNF complex gene variations are associated with a higher tumor mutational burden and a better response to immune checkpoint inhibitor treatment: A pan-cancer analysis of next-generation sequencing data corresponding to 4591 cases. Cancer Cell Int..

[78] Gillet N., Dumont E., Bignon E. (2024). DNA damage and repair in the nucleosome: Insights from computational methods. Biophys. Rev..

[79] Ozair A., Bhat V., Alisch R.S., Khosla A.A., Kotecha R.R., Odia Y., McDermott M.W., Ahluwalia M.S. (2023). DNA methylation and histone modification in low-grade gliomas: Current understanding and potential clinical targets. Cancers.

[80] Mehrmohamadi M., Sepehri M.H., Nazer N., Norouzi M.R. (2021). A comparative overview of epigenomic profiling methods. Front. Cell Dev. Biol..

[81] Manna S., Mishra J., Baral T., Kirtana R., Nandi P., Roy A., Chakraborty S., Niharika, Patra S.K. (2023). Epigenetic signaling and crosstalk in regulation of gene expression and disease progression. Epigenomics.

[82] Reddington J.P., Perricone S.M., Nestor C.E., Reichmann J., Youngson N.A., Suzuki M., Reinhardt D., Dunican D.S., Prendergast J.G., Mjoseng H. (2013). Redistribution of H3K27me3 upon DNA hypomethylation results in de-repression of Polycomb target genes. Genome Biol..

[83] Pan M.R., Hsu M.C., Chen L.T., Hung W.C. (2018). Orchestration of H3K27 methylation: Mechanisms and therapeutic implication. Cell Mol. Life Sci..

[84] Pettini F., Visibelli A., Cicaloni V., Iovinelli D., Spiga O. (2021). Multi-omics model applied to cancer genetics. Int. J. Mol. Sci..

[85] Latini A., Borgiani P., Novelli G., Ciccacci C. (2019). miRNAs in drug response variability: Potential utility as biomarkers for personalized medicine. Pharmacogenomics.

[86] Jensen S.Ø., Øgaard N., Ørntoft M.B.W., Rasmussen M.H., Bramsen J.B., Kristensen H., Mouritzen P., Madsen M.R., Madsen A.H., Sunesen K.G. (2019). Novel DNA methylation biomarkers show high sensitivity and specificity for blood-based detection of colorectal cancer—A clinical biomarker discovery and validation study. Clin. Epigenet..

[87] Zhang Z., Wang G., Li Y., Lei D., Xiang J., Ouyang L., Wang Y., Yang J. (2022). Recent progress in DNA methyltransferase inhibitors as anticancer agents. Front. Pharmacol..

[88] Rehman S.U., Abdullah M., Khan Z.K., Shurovi M. (2025). The role of DNA methylation and histone modifications in the pathogenesis of hematological malignancies and solid cancers: Mechanisms, clinical implications, and therapeutic potential. Asian J. Med. Biol. Res..

[89] Clarke K., Young C., Liberante F., McMullin M.F., Thompson A., Mills K. (2019). The histone deacetylase inhibitor Romidepsin induces a cascade of differential gene expression and altered histone H3K9 marks in myeloid leukaemia cells. Oncotarget.

[90] Zhang L., Li H.T., Shereda R., Lu Q., Weisenberger D.J., O’Connell C., Machida K., An W., Lenz H.-J., El-Khoueiry A. (2022). DNMT and EZH2 inhibitors synergize to activate therapeutic targets in hepatocellular carcinoma. Cancer Lett..

[91] Ramalho-Carvalho J., Fromm B., Henrique R., Jeronimo C. (2016). Deciphering the function of non-coding RNAs in prostate cancer. Cancer Metastasis Rev..

[92] Alizadeh M., Safarzadeh A., Beyranvand F., Ahmadpour F., Hajiasgharzadeh K., Baghbanzadeh A., Baradaran B. (2019). The potential role of miR-29 in health and cancer diagnosis, prognosis, and therapy. J. Cell Physiol..

[93] Su X., Yan L., Si J., Wang Z., Liang C., Peng K., Shen J., Duan S. (2024). *LINC00319*: Unraveling the spectrum from gene regulation to clinical applications in cancer progression. Gene.

[94] Yuan Y., Tang Y., Fang Z., Wen J., Wicha M.S., Luo M. (2025). Long non-coding RNAs: Key regulators of tumor epithelial/mesenchymal plasticity and cancer stemness. Cells.

[95] Hernández-Lemus E., Ochoa S. (2024). Methods for multi-omic data integration in cancer research. Front. Genet..

[96] Santiago-Rodriguez T.M., Hollister E.B. (2021). Multi ‘omic data integration: A review of concepts, considerations, and approaches. Semin Perinatol..

[97] Desaulniers D., Vasseur P., Jacobs A., Aguila M.C., Ertych N., Jacobs M.N. (2021). Integration of epigenetic mechanisms into non-genotoxic carcinogenicity hazard assessment: Focus on DNA methylation and histone modifications. Int. J. Mol. Sci..

[98] Papanicolau-Sengos A., Aldape K. (2022). DNA methylation profiling: An emerging paradigm for cancer diagnosis. Annu. Rev. Pathol..

[99] Kundu S., Das R., Laskar S., Choudhury Y., Ghosh S.K. (2020). Principles of bi-sulfite conversion of DNA and methylation-specific PCR (MSP) in biological research. Epigenetics Methods.

[100] Pidsley R., Zotenko E., Peters T.J., Lawrence M.G., Risbridger G.P., Molloy P., van Dijk S., Mühlhäusler B., Stirzaker C., Clark S.J. (2016). Critical evaluation of the Illumina MethylationEPIC BeadChip microarray for whole-genome DNA methylation profiling. Genome Biol..

[101] Lin N., Liu J., Castle J., Wan J., Shendre A., Liu Y., Wang C., He C. (2021). Genome-wide DNA methylation profiling in human breast tissue by Illumina TruSeq methyl capture EPIC sequencing and Infinium MethylationEPIC BeadChip microarray. Epigenetics.

[102] Klemm S.L., Shipony Z., Greenleaf W.J. (2019). Chromatin accessibility and the regulatory epigenome. Nat. Rev. Genet..

[103] Sepehri Z., Beacon T.H., Osman F.D., Jahan S., Davie J.R. (2019). DNA methylation and chromatin modifications. Nutritional Epigenomics.

[104] Kumar B., Navarro C., Yung P.Y.K., Lyu J., Mantero A.S., Katsori A.-M., Schwämmle H., Martin M., Elsässer S.J. (2025). Multiplexed chromatin immunoprecipitation sequencing for quantitative study of histone modifications and chromatin factors. Nat. Protoc..

[105] Fosslie M., Manaf A., Lerdrup M., Hansen K., Gilfillan G.D., Dahl J.A. (2020). Going low to reach high: Small-scale ChIP-seq maps new terrain. Wiley Interdiscip. Rev. Syst. Biol. Med..

[106] Akdogan-Ozdilek B., Duval K.L., Meng F.W., Murphy P.J., Goll M.G. (2022). Identification of chromatin states during zebrafish gastrulation using CUT&RUN and CUT&Tag. Dev. Dyn..

[107] Tsoneva D.K., Ivanov M.N., Conev N.V., Manev R., Stoyanov D.S., Vinciguerra M. (2023). Circulating histones to detect and monitor the progression of cancer. Int. J. Mol. Sci..

[108] Baumann A.A., Buribayev Z., Wolkenhauer O., Salybekov A.A., Wolfien M. (2025). Epigenomic echoes—Decoding genomic and epigenetic instability to distinguish lung cancer types and predict relapse. Epigenomes.

[109] Micheel J., Safrastyan A., Wollny D. (2021). Advances in non-coding RNA sequencing. Noncoding RNA.

[110] Dai R., Wang Z., Ahmed S.A. (2021). Epigenetic contribution and genomic imprinting Dlk1-Dio3 miRNAs in systemic lupus erythematosus. Genes.

[111] Nguyen T.T., Suman K.H., Nguyen T.B., Nguyen H.T., Do D.N. (2022). The role of miR-29s in human cancers—An update. Biomedicines.

[112] de Gonzalo-Calvo D., Marchese M., Hellemans J., Betsou F., Frisk N.L.S., Dalgaard L.T., Lakkisto P., Foy C., Scherer A., García-Berrojo M.L. (2022). Consensus guidelines for the validation of qRT-PCR assays in clinical research by the CardioRNA consortium. Mol. Ther. Methods Clin. Dev..

[113] Garbo E., Del Rio B., Ferrari G., Cani M., Napoli V.M., Bertaglia V., Capelletto E., Rolfo C., Novello S., Passiglia F. (2023). Exploring the potential of non-coding RNAs as liquid biopsy biomarkers for lung cancer screening: A literature review. Cancers.

[114] Toden S., Goel A. (2022). Non-coding RNAs as liquid biopsy biomarkers in cancer. Br. J. Cancer.

[115] Dagogo-Jack I., Shaw A.T. (2018). Tumour heterogeneity and resistance to cancer therapies. Nat. Rev. Clin. Oncol..

[116] Carter B., Zhao K. (2021). The epigenetic basis of cellular heterogeneity. Nat. Rev. Genet..

[117] Mukherjee A., Abraham S., Singh A., Balaji S., Mukunthan K. (2025). From data to cure: A comprehensive exploration of multi-omics data analysis for targeted therapies. Mol. Biotechnol..

[118] Lin L., Liu Y. (2025). Advances in epigenomic sequencing and their applications in cancer diagnostics. Precis. Chem..

[119] Cabezón M., Malinverni R., Bargay J., Xicoy B., Marcé S., Garrido A., Tormo M., Arenillas L., Coll R., Borràs J. (2021). Different methylation signatures at diagnosis in patients with high-risk myelodysplastic syndromes and secondary acute myeloid leukemia predict azacitidine response and longer survival. Clin. Epigenet..

[120] Kagan A.B., Garrison D., Anders N.M., Webster J.A., Baker S.D., Yegnasubramanian S., Rudek M.A. (2023). DNA methyltransferase inhibitor exposure response: Challenges and opportunities. Clin. Transl. Sci..

[121] Zhang H., Pang Y., Yi L., Wang X., Wei P., Wang H., Lin S. (2025). Epigenetic regulators combined with tumour immunotherapy: Current status and perspectives. Clin. Epigenet..

[122] Cross M., Bach E., Tran T., Krahl R., Jaekel N., Niederwieser D., Junghanss C., Maschmeyer G., Al-Ali H.K. (2013). Pretreatment long interspersed element (LINE)-1 methylation levels, not early hypomethylation under treatment, predict hematological response to azacitidine in elderly patients with acute myeloid leukemia. OncoTargets Ther..

[123] Müller D., Győrffy B. (2022). DNA methylation-based diagnostic, prognostic, and predictive biomarkers in colorectal cancer. Biochim. Biophys. Acta Rev. Cancer.

[124] Ponomaryova A.A., Rykova E.Y., Gervas P.A., Cherdyntseva N.V., Mamedov I.Z., Azhikina T.L. (2020). Aberrant methylation of LINE-1 transposable elements: A search for cancer biomarkers. Cells.

[125] Voso M.T., Santini V., Fabiani E., Fianchi L., Criscuolo M., Falconi G., Guidi F., Hohaus S., Leone G. (2014). Why methylation is not a marker predictive of response to hypomethylating agents. Haematologica.

[126] Perrier A., Didelot A., Laurent-Puig P., Blons H., Garinet S. (2020). Epigenetic mechanisms of resistance to immune checkpoint inhibitors. Biomolecules.

[127] Wong K.K., Lawrie C.H., Green T.M. (2019). Oncogenic roles and inhibitors of DNMT1, DNMT3A, and DNMT3B in acute myeloid leukaemia. Biomark. Insights.

[128] Liu P., Yang F., Zhang L., Hu Y., Chen B., Wang J., Su L., Wu M., Chen W. (2022). Emerging role of different DNA methyltransferases in the pathogenesis of cancer. Front. Pharmacol..

[129] Yu Y., Fu W., Xie Y., Jiang X., Wang H., Yang X. (2024). A review on recent advances in assays for DNMT1: A promising diagnostic biomarker for multiple human cancers. Analyst.

[130] Jones P., Issa J.P., Baylin S. (2016). Targeting the cancer epigenome for therapy. Nat. Rev. Genet..

[131] Song J., Yang P., Chen C., Ding W., Tillement O., Bai H., Zhang S. (2025). Targeting epigenetic regulators as a promising avenue to overcome cancer therapy resistance. Signal Transduct. Target. Ther..

[132] Chervona Y., Costa M. (2012). Histone modifications and cancer: Biomarkers of prognosis?. Am. J. Cancer Res..

[133] McGarvey K.M., Fahrner J.A., Greene E., Martens J., Jenuwein T., Baylin S.B. (2006). Silenced tumor suppressor genes reactivated by DNA demethylation do not return to a fully euchromatic chromatin state. Cancer Res..

[134] Suárez B., Solé C., Márquez M., Nanetti F., Henderson Lawrie C.H. (2022). Circulating MicroRNAs as Cancer Biomarkers in Liquid Biopsies.

[135] Marcucci G., Radmacher M.D., Maharry K., Mrózek K., Ruppert A.S., Paschka P., Vukosavljevic T., Whitman S.P., Baldus C.D., Langer C. (2008). MicroRNA expression in cytogenetically normal acute myeloid leukemia. N. Engl. J. Med..

[136] Kubota H., Ueno H., Tasaka K., Isobe T., Saida S., Kato I., Umeda K., Hiwatari M., Hasegawa D., Imamura T. (2024). RNA-seq-based miRNA signature as an independent predictor of relapse in pediatric B-cell acute lymphoblastic leukemia. Blood Adv..

[137] Zsidó B.Z., Hetényi C. (2020). Molecular structure, binding affinity, and biological activity in the epigenome. Int. J. Mol. Sci..

[138] Liu M.C., Oxnard G.R., Klein E.A., Swanton C., Seiden M.V., CCGA Consortium (2020). Sensitive and specific multi-cancer detection and localization using methylation signatures in cell-free DNA. Ann. Oncol..

[139] Bai J., Jiang P., Ji L., Lam W.K.J., Zhou Q., Ma M.L., Ding S.C., Ramakrishnan S., Wan C.W., Yang T.C. (2024). Histone modifications of circulating nucleosomes are associated with changes in cell-free DNA fragmentation patterns. Proc. Natl. Acad. Sci. USA.

[140] Martino M.T., Tagliaferri P., Tassone P. (2025). MicroRNA in cancer therapy: Breakthroughs and challenges in early clinical applications. J. Exp. Clin. Cancer Res..

[141] Grishina O., Schmoor C., Döhner K., Hackanson B., Lubrich B., May A.M., Cieslik C., Müller M.J., Lübbert M. (2015). DECIDER: Prospective randomized multicenter phase II trial of low-dose decitabine administered alone or in combination with the histone deacetylase inhibitor valproic acid and all-trans retinoic acid in patients > 60 years with acute myeloid leukemia who are ineligible for induction chemotherapy. BMC Cancer.

[142] Sun Z., Wang X., Vedell P., Kocher J.P. (2022). DNA methylation signature predicts cancer response to demethylation agents from profiling diverse cancer cell lines. Cancer Commun..

[143] Javorniczky N.R., Grishina O., Hund I., Pantic M., Pfeifer D., Schmoor C., Thomas J., Duyster J., Becker H., Lübbert M. (2023). Long-term decitabine/retinoic acid maintenance treatment in an elderly sAML patient with high-risk genetics. Clin. Epigenet..

[144] Yang T., Yang Y., Wang Y. (2021). Predictive biomarkers and potential drug combinations of epi-drugs in cancer therapy. Clin. Epigenet..

[145] Dombret H., Seymour J.F., Butrym A., Wierzbowska A., Selleslag D., Jang J.H., Kumar R., Cavenagh J., Schuh A.C., Candoni A. (2015). International phase 3 study of azacitidine vs conventional care regimens in older patients with newly diagnosed AML with >30% blasts. Blood.

[146] DiNardo C.D., Jonas B.A., Pullarkat V., Thirman M.J., Garcia J.S., Wei A.H., Konopleva M., Döhner H., Letai A., Fenaux P. (2020). Azacitidine and Venetoclax in Previously Untreated Acute Myeloid Leukemia. N. Engl. J. Med..

[147] Baretti M., Karunasena E., Zahurak M., Walker R., Zhao Y., Pisanic T.R., Wang T.H., Greten T.F., Duffy A.G., Gootjes E. (2021). A phase 2 trial of gemcitabine and docetaxel in patients with metastatic colorectal adenocarcinoma with methylated checkpoint with forkhead and ring finger domain promoter and/or microsatellite instability phenotype. Clin. Transl. Sci..

[148] Crabb S.J., Danson S., Catto J.W.F., Hussain S., Chan D., Dunkley D., Downs N., Marwood E., Day L., Saunders G. (2021). Phase I trial of DNA methyltransferase inhibitor guadecitabine combined with cisplatin and gemcitabine for solid malignancies including urothelial carcinoma (SPIRE). Clin. Cancer Res..

[149] Baylin S.B., Jones P.A. (2011). A decade of exploring the cancer epigenome—Biological and translational implications. Nat. Rev. Cancer.

[150] Wisman G.B.A., Wojdacz T.K., Altucci L., Rots M.G., DeMeo D.L., Snieder H. (2024). Clinical promise and applications of epigenetic biomarkers. Clin. Epigenet..

[151] Guo M., Peng Y., Gao A., Du C., Herman J.G. (2019). Epigenetic heterogeneity in cancer. Biomark. Res..

[152] Horgan D., Hamdi Y., Lal J.A., Nyawira T., Meyer S., Kondji D., Francisco N.M., De Guzman R., Paul A., Bernard B. (2023). Framework for adoption of next-generation sequencing (NGS) globally in the oncology area. Healthcare.

